# Modified strip tillage blades for two-wheel tractor seed drills improves maize crop establishment under conservation agriculture

**DOI:** 10.1016/j.deveng.2021.100061

**Published:** 2021

**Authors:** Muhammad Arshadul Hoque, Mahesh K. Gathala, Md Mosharraf Hossain, A.T.M. Ziauddin, Timothy J. Krupnik

**Affiliations:** a Farm Machinery and Postharvest Engineering Division, Bangladesh Agricultural Research Institute (BARI), Gazipur, 1701, Bangladesh; b Department of Farm Power and Machinery, Bangladesh Agricultural University, Mymensingh, 2202, Bangladesh; c International Maize and Wheat Improvement Center (CIMMYT), Sustainable Intensification Program, House 10/B, Road 53, Gulshan-2, Dhaka, 1213, Bangladesh

**Keywords:** Two-wheeled tractor, Strip tillage, Soil furrow, Emergence rate index, Furrow soil backfill

## Abstract

Two-wheel tractors (2WTs) are widely used by resource-poor farmers to prepare land in the Eastern Indo-Gangetic Plains (EIGP). This paper demonstrates that improved tillage blade design can enhance maize crop establishment under strip tillage, which falls under the rubric of conservation agriculture (CA). In order to achieve this aim, it is necessary to identify appropriate blade design and rotational speed for power tiller operated seeders, or PTOS, which can be attached to 2WTs and that are increasingly popular in the EIGP. We conducted experiments over two years in two locations in the EIGP within Bangladesh with loam and clay loam soils, respectively. Four blades designed with varying tip angles and five levels of rotational speed were compared with commercially available C-shaped blades sold with 2WTs. Torque and power requirements for strip tillage decreased with decreasing blade tip angle and rotational speed. The best combination of blade design and rotational speed was found with a 15° blade tip angle at 320 RPM. This combination resulted in higher furrow cross sectional area, more soil backfill with appropriately sized soil aggregates, and better seeding depth than C-shaped and 45° tip angle blades. These characteristics also facilitated improved crop establishment on both soil types. Our results indicate that strip-till maize establishment can be improved in Bangladesh by substituting commercially-available C-shaped blades with a 15° blade tip angle at appropriate 320 RPM, though machinery operators will require educational efforts to learn how to fine-tune RPM to improve crop establishment and achieve more sustainable crop establishment systems.

## Highlights

• Significant highlights of this research:
• Increase potential use of 2WTs in conservation agriculture-based crop production in Eastern Gangetic Plains.
• Reduce in torque and power requirement of strip tillage seeders.
• Improve in furrow backfill, uniform seed depth and better crop establishment.
• Increase conservation agriculture adoption by smallholders.

## 1 Introduction

Power tillers, often referred to as two-wheeled tractors (2WTs), are popular for land preparation in smallholder farming systems in South and East Asia including in Bangladesh, eastern India, the Terrai of Nepal, as well as in parts of Myanmar, Thailand, Vietnam, South Korea and China (Chertkiattipol and Niyamapa, 2010; Siddique et al., 2012). Of these, Bangladesh is the most impoverished and densely populated country with an average of 1240 people km^−2^ (World Bank 2018a). More than 66 million people in Bangladesh subsist and earn their income from agriculture (World Bank 2018b), with an average farm size of less than half a hectare (Hossain et al. 2007). Monsoon season rice cropping is common in Bangladesh, with farmers commonly rotating rice with winter season maize or wheat (Gathala et al., 2016, 2017). More than 700,000 2WT attachable power tillers are used by smallholder farmers in Bangladesh for land preparation (Rahman et al., 2017). This usually involves multiple passes of a power tiller with slow rotational speed (200–250 RPM) for seedbed preparation, followed by manual broadcasting or hand planting of seeds in lines (Ladha et al., 2009; Hoque and Miah, 2015).

**Fig. 1:**
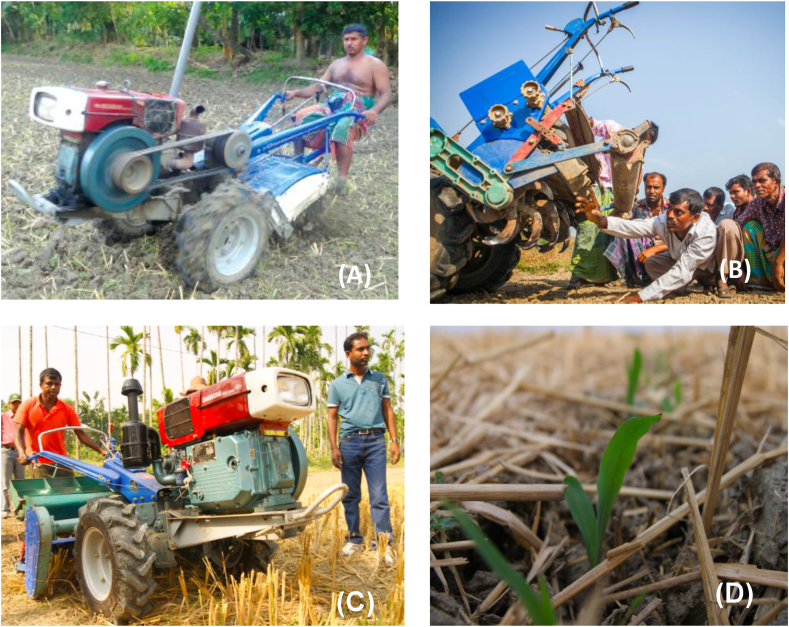
(A) A Chinese manufactured two-wheel tractor power tiller used for conventional tillage in Bangladesh. (B) Detail of farmers examining the rear portion of a power-tiller operated seeder (PTOS) with conventional C-shaped blades in Bangladesh. (C) A PTOS attached to a two-wheel tractor being used to seed maize by strip tillage (note the anchored rice residue). (D) The resulting emerging maize crop. Photo A and B by R. Martin. Photos B and C by TJ Krupnik.

So-called ‘power tiller operated seeders’ (PTOS), that are commercially available in China and widely sold on the Bangladeshi market, present an alternative. By decoupling and removing the conventional power tiller from the power take-off unit, farmers can re-attach a PTOS to a 2WT, which utilizes a high-speed rotovator, 48 tillage blades, and fluted roller seeding system. By using the PTOS, farmers can achieve land preparation and seeding simultaneously while also reducing the number of tillage operations to from two or more to one (Fig. 1). Each of the blades on a PTOS are bent and C-shaped. The rotovator of the PTOS typically runs at 480–500 RPM to cultivate a width of 1.2 m. A single PTOS can plant a maximum of six 20 cm interval rows, although farmers can also make use of a lower number of fluted-roller seeding units to establish more widely spaced crops. This is the case for crops like maize that is typically established at 60 cm row interval (Matin et al., 2014).

The PTOS is versatile in use and can assist in the preparation of soil through tillage. It also simultaneously places seed and fertilizer at the right depth with adjustable mounted furrow openers. In addition, the PTOS has a following roller that presses the soil after seeding for better seed to soil contact. The PTOS can also be easily converted for strip tillage by removing the significant total number of blades, with an appropriate adjustment to desire seeding depth and spacing (Hoque and Miah, 2015). By removing a desire number of the detachable blades from the rotovator system, it becomes possible to directly drill seed into 3–5 cm wide furrows with the soil between crop rows left untilled (Krupnik et al. 2013; Roy et al., 2009; Haque et al., 2013). This procedure of placing seed into narrow furrows and limiting soil inversion can be referred to as strip tillage (FAO, 2000), and can be utilized with conservation agriculture (CA) based tillage and crop establishment systems that minimize soil disturbance, maintain soil cover with living or dead mulches, and employ crop rotations (Gathala et al., 2015, 2016).

A range of farm machinery types have been developed in Bangladesh for CA, with research indicating the potential for production cost savings, improved water use efficiency, reduced irrigation requirements, and improved soil quality with environmental benefits, among others (Hossain et al., 2015, Roy et al., 2009; Matin et al., 2008; Islam et al., 2019; Haque et al., 2011; Gathala et al., 2020). When used in combination with mulching and crop rotation, PTOS aided strip tillage can be considered a conservation agriculture (CA) practice (Hossain et al., 2015; Gathala et al., 2015, 2016; Islam et al., 2019; Hoque and Miah, 2015). A number of studies have evaluated PTOS-based strip tillage of wheat, maize, mungbean and chickpea, but with varying results depending on the crop, soil type, and degree of soil moisture, among other factors (Esdaile, 2010; Hossain et al., 2009; Hoque and Karim, 2015; Krupnik et al. 2014; Gathala et al., 2020). However, technical improvements in PTOS engineering are needed to arrive at optimal blade designs, rotary speed, and suitable furrow openers with an appropriate seed metering systems to optimize crop establishment and assure efficient and low-cost strip-till seed drills that can be accessible to farmers in Bangladesh (Krupnik et al., 2013; Siddique et al., 2012).

In addition to weed control, the primary purpose of soil tillage is to create a suitable seedbed for optimal crop emergence and establishment. Improved strip-till seeding and crop establishment is likely to require the redesign and fine-tuning of bent-shape rotary blades to limit soil throwing outside furrows (Beeny and Khoo, 1970), while also creating improved furrow architecture and assuring adequate soil infill to avoid seed predation and desiccation. Combined with achieving the desired rate of seed germination and early seedling vigor, these aspects are crucial indications of seedbed quality (Matin et al., 2015). A rule of thumb for appropriate soil coverage is to provide soil particle infill that is 3–4 times the seed diameter within furrows (Dekalb, 2009).

Improper tillage blade design and configuration is a major constraint for strip tillage that can result in poor crop establishment and lower yields (Temesgen et al., 2007). Most commercially available PTOS used for strip tillage are produced in China and come with bent C-shaped rotary blades (Krupnik et al. 2013). In studies using these blades in Bangladesh, both Haque et al. (2010) and Matin et al. (2017) concluded that overly narrow and shallow strip tillage is less effective in heavier soils that remain overly moist, or in quick drying soils where seed placed within strips can desiccate and die. Poor backfill has also been observed with four-wheeled tractor based rotary strip tillage using L- and C-shapes blades (Lee et al., 2003). While Hossain et al. (2009) found that using conventional C-shaped rotary blades can produce a seedbed of fine tilth, the high-speed rotary action of the PTOS can scatter soil out of furrows, hampering crop establishment.

The performance of different blades varies as a function of tillage torque, impact force and specific tilling energy through rotary speed (Lee et al., 2003; Chertkiattipol and Niyamapa, 2010). Rotavators usually have ‘L’ shape, ‘C’ shape, or ‘J’ shaped blades. Hook tines and straight knife blades can also be used for different seeding goals and soil types. Depending on the soil conditions, blade geometry and velocity ratio may result in different soil aggregation and power consumption. The need for improved design of blades for strip tillage has therefore been flagged as an area of research importance (Krupnik et al., 2013; Mahal et al., 2012; Matin et al., 2017).

In addition to modifications in blade shape, changes in rotavator speed may also affect improved crop establishment by maintaining a small bite length at effective operating travel speeds, while also achieving the desired level of soil pulverization and furrow in-fill. Higher rotary speeds and decreasing bite length results in more uniform depth along the furrow (Celik and Altikat, 2008), but also increases soil acceleration and evacuation from the furrow. Soil cutting by a rotary blade also results in soil compression, compaction and cracking where the blade tip contacts with the furrow (Chertkiattipol and Niyamapa, 2010; Matin et al. 2014). These are issues that should be addressed when redesigning blades.

Another problem associated with the use of rotary blades for strip tillage is seedbed creation. Soil furrow infill aggregate size must be fine enough to assure moisture retention and optimal seed-to-soil contact for optimum germination and early growth. Optimum soil tilth, measured by mean aggregate diameter for wheat and maize, both of which are widely sown after the monsoon season rice crop in Bangladesh, is 1–2 mm and 5 mm in loam soils, respectively (Braunack and Dexter, 1989). The degree of clod breaking to achieve optimum soil aggregates depends on tillage implements and rotary tillage (Nage et al., 2011).

Matin et al. (2014) modified conventional blades to reduce the width of the sidelong sections to 22 mm and designated half-width blades. In a laboratory setting and without anchored residue or mulch, 74% furrow backfill was obtained by reducing the soil carrying and throwing by straighter blades at 500 RPM. Similar results were demonstrated by Matin et al. (2021) subsequently reconfirmed similar results in a soil bin study using moist clay soils from southern Bangladesh. There is however currently no evidence on the performance of modified blades under on-farm settings in South Asia when winter season crops are established by drilling seed into furrows through anchored residue of the previous monsoon season rice crop. This paper responds by providing evidence of the importance of blade design for maize crop establishment under strip tillage using a 2WT driven PTOS under on-farm conditions, including loam and clay loam soils with anchored rice residue as a mulch over two years.

## 2 Materials and methods

### 2.1 Blade design

**Fig. 2:**
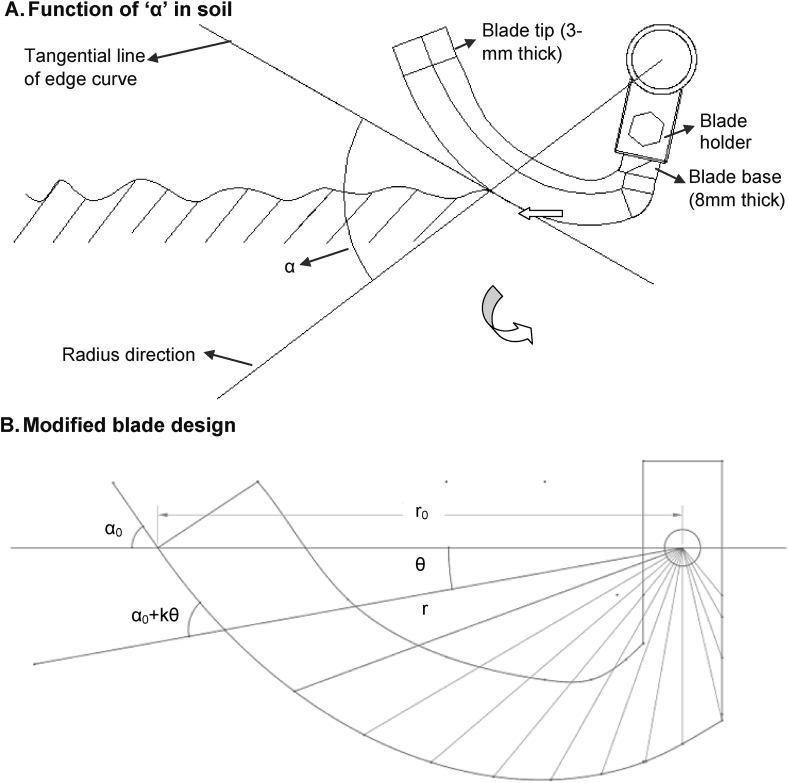
Working functions of blade in soil and modified blade design for strip tillage. (A) Working function of ‘α’ in soil contact. (‘α_0_’ is the edge-curve angle of the blade for soil cutting; K is constant, θ is blade rotational degree and *r* is the radius of rotating blade), (B) Modified blade design.

When a rotary blade cuts the soil, the angle α between the radius direction of the turning blade and the tangential line of its edge-curve (Fig. 2A) are the main points to be considered in blade design (Sakai, 1978a, 1978b). The straighter lengthwise portions of the blade typically increase the cutting ability of weeds, straw residues, and the soil itself when α is small. If α is small, the area of the blade that contacts with the soil to form furrows will also be narrow. This results in reduced friction area between the soil and blade, thereby lowering lower tillage resistance (Sakai, 1978a, 1978b).

Five new blade types were designed, fabricated and configured for tillage evaluation at Mahbub Engineering and the Bangladesh Agricultural Research Institute (BARI) in Jamalpur, Bangladesh (Table 1). We designed new blades utilizing Equation (1) following Sakai (1978a, 1978b),

(1) $$r=r_{0}Sin^{\frac{1}{k}}\alpha_{0}Sin^{-\frac{1}{k}}\left(\alpha_{0}+k\theta \right)$$

where *r* is the calculated radius of the rotating blade spiral that depends on the variable *θ* in the function of blade cutting edge-curve, with *r*_0_ signifying the maximum radius at the tip of the edge-curve as shown in Fig. 2B α_0_ is the edge-curve angle at r_0_, and *k* is a constant to give changing angles from α functions. If an increase of 10° is expected within the range of 180°, *k* is assigned a value of 10°/180° or 1/18 for all angles.

**Table 1:** Comparative Characteristics of different blades used in experiments. C indicates the Chinese traditional blade, while MB indicates the modified blade with 45°, 30°, 15° and 0° tip rake angles.

Characters	Blade shapes, top rake angles and additional details				
	C 48° angle	MB 45° angle	MB 30° angle	MB 15° angle	MB 0° angle
Sidelong length (mm)	65	18	18	18	18
Cut width (mm)	46.00	24.81	20.47	15.42	8.00
Blade radius (mm)	159	160	160	160	160
Inter-blade space during strip tillage (‘ɗ’ mm)	0	10.39	19.06	29.16	40.00
Blade image					

Modified blades were designed considering *r*_0_ = 160 mm since conventionally Chinese blades that typically come with commercially available PTOS machines have this curvature radius. To reduce the potential for blades to displace or even hook and carry straw residues when used for strip tillage, α was taken as 55°. This configuration should result in a about one-third of blade length from the tip to base carrying no straw (Sakai, 1978a, 1978b). An increase of 10° and *k* = 1/18 and its effect on *r* is provided in Fig. 2B. The thickness of the new blade was 8 mm in the holder section which gradually decreases to 3 mm at the end of tip. The cutting edge of the blades was tapered from 8 mm to 3 mm, creating a sharp edge for furrow formation. The blades were fabricated with carbon steel (AISI 1180) as designed and drawn in Solid Works (Version, 2014 × 64, Dassault Systèmes, Waltham, MA). Five types of blades, such as Conventional C-shaped blades with 48°, modified curved blade with 45, 30, 15 and 0° tip angles were subsequently manufactured.

### 2.2 Experimental design and crop establishment

#### 2.2.1 Experimental layout

Field experiments using were conducted over two seasons (two maize seasons) at the BARI Regional Agricultural Research Stations in Jamalpur (22° 47′ 22.2432″N, 90° 17′ 23.7228″E) and Barishal (24° 56′ 30.6844″N, 89° 55′ 40.3882″E) in the winter ‘*rabi*’ season in 2013–14 and 2014–15. The soils of Jamalpur and Barishal are loam and clay loam, respectively. Soil moisture and bulk density of the topsoil (0–15 cm) at Jamalpur in first season were 24.50% and 1.42 g cm^−3^. In the second season, they were 23.47% and 1.42 g cm^−3^, respectively. At Barishal, soil moisture and soil bulk density were 25.31% and 1.33 g cm^−3^ and 27.01% and 1.32 g cm^−3^ in 2013 and 2014, respectively. Experiments were laid out in a split-plot design with four replications, with the main plot as rotating blade speed (240, 320, 400, 480 and 560 RPM) and the sub-plot allocated to five different blade shapes, including conventional (C) and modified blade (MB) designs as C48, MB45, MB30, MB15 and MB0, respectively. The smallest individual plot size was 10 m × 2.5 m.

#### 2.2.2 Power-tiller operated seeder configuration

On a PTOS, shaft holders are fixed in a spiral configuration. During remounting of strip tillage blades, care was taken to arrange them in face to face. Conventional C-shaped blades were spaced in such a way that width of cut of the right blade overlapped with the cut of the left blade (Fig. 4A). Width of the modified blades was conversely less than C-shaped blades (Fig. 4B), which there was an inter blade space arrangement if used for strip tillage. As the traditional PTOS is designed for full rotovator tillage, they have 48 blade holders. When converted to strip tillage for maize planting, only a portion of the holders are used to assure two strip furrow arrangements at 60 cm row spacing (Fig. 4C). Blades were accordingly arranged face to face in adjacent four holders for 6 cm wide strip, and operated at speeds of 560, 480, 400, 320, 240 RPM. Rotary blade shaft speed was modified by changing the chain sprocket wheel size combination. During experimentation, speeds were verified with a digital Tachometer (PROVA, RM-1500, 10–99,990 RPM, accuracy 0.04% ± 2). If needed, speed was adjusted by varying the engine speed. Average operational forward speed was 2.5 km h^−1^ as it was described optimum speed for two-wheel tractor operated seeders (Solomon, 2017).

#### 2.2.3 Crop establishment and management

In Bangladesh, maize is usually grown after and rotated with monsoon season rice. As such, in the season preceding the 2013–14 winter maize crop, a monsoon season rice crop was transplanted at a density of 20 × 20 cm and managed uniformly across all plots. After harvest, 25 cm height of anchored rice stubble was retained as recommended for CA with at least 30% of the soil covered with residue (Gathala et al., 2015). Five days before planting, glyphosate was applied at 3.7 L ha^−1^ (01 kg a. i. Ha^−1^) using 320–400 L ha^−1^ of water with a calibrated three-nozzle flat-fan spray boom. All plots were sown by the same experienced PTOS operator in both locations. The basal fertilizer dose in both locations for maize was 80 kg nitrogen, 45 kg P_2_O_5_, 65 kg K_2_O ha^−1^. Diammonium Phosphate (DAP) was applied at 50 kg ha^−1^ by PTOS drilling with seed at sowing. A BARI designed inclined plate PTOS (Hoque and Miah, 2015), which has T-inverted furrow openers, was used to plant hybrid NK40 maize seed at a seed-to-seed spacing of 20 cm in rows, and 60 cm between row intervals. Sowing was done on 10 November in 2013 and November 14, 2014 at Jamalpur. In Barishal, sowing took place on 5 April in 2013 and March 10, 2014 as access in the field was only possible in February onwards depending on season due to prolonged excess soil moisture following the retreat of the monsoon. Twenty-five days after sowing, light irrigation was applied in both locations. Although experimental measurements were terminated at 30 days after sowing, the maize crop was grown to maturity using uniform management across treatments after which an identical monsoon season rice crop was rotated before the second season of maize establishment.

### 2.3 Data collection and measurements

#### 2.3.1 Top width and cross-sectional area of tilled strips

The top width of the furrows was measured from ten randomly selected locations within each replication immediately after sowing with a steel slide caliper. To determine cross sectional area, the volume of a randomly selected 25 cm long furrow section was measured using the sand replacement method (Matin et al., 2014). Well graded, air-dried coarse sand was used for these measurements. Bulk density (Bd) of the used sand at Barishal was 1.5 and 1.48 g cm^−3^ during 2013–14 and 2014–15 respectively, and at Jamalpur the Bd was 1.44 g cm^−3^ in both the seasons. Volume of the selected section was divided with the length of the section to identify the average cross-sectional area of the furrow.

#### 2.3.2 Furrow soil backfill

Furrow backfill was measured as the gravimetric proportion of soil retained within the furrow immediately after sowing. All loose soil remaining in the furrow in a 25 cm section was collected using a wooden scoop and fiber brush from ten randomly selected places of each plot. Dry mass of the samples was determined by the oven method at 105 ^°^C for 48 h (Park et al., 2002). The corresponding furrow volume was measured using the sand replacement method as described in 2.2.1. Percent furrow soil backfill (*F*_*b*_) was calculated according to Equation (2),

(2) $$F_{b}=\frac{W_{d}}{V\rho }\times 100$$

where *W*_*d*_ is total dry soil mass (g) remaining in the furrow, *V* is volume of the tilled furrow (cm^3^), and *ρ* is bulk density (dry weight basis) of the untilled soil (g cm^−3^). Using this method, a 100% backfill result indicates the entire amount of soil originating from the furrow volume remained within the furrow boundaries during tillage.

#### 2.3.3 Proportion of soil aggregates

To determine soil particle size distribution, soil samples were collected from three randomly selected squares of 0.25 m^2^ (50 by 50 cm) area. A wooden square box (0.25 m^2^) was placed on the center of furrow to cover both side equal distance, after which all loose soil particles were collected by using a brush and scoop. Particles were retained in polythene bags taken to the laboratory and air dried to constant weight. All soil samples were then weighed with a 0.01 g precision scale and manually sieved at a consistent rhythm for 60 s using eight sieves (mesh sizes of 63, 32, 16, 8, 4, 2 and 1 mm). The amount of remaining soil particles on each sieve were then re-weighed. Aggregates 1–8 mm diameter were considered to as the optimum particle size for maize seedbed formation following Celik and Alikat (2010). The proportion of soil aggregates in large, optimum and smaller groups were determined following equation (3),

(3) $$SA=\frac{S_{s}}{S_{t}}\times 100$$

where *SA* is the percentage of aggregates in size categories by mass, *S*_*s*_ is weight (g) of the air dried sample retain in each group, and *S*_*t*_ is total weight (g) of the air dried sample.

#### 2.3.4 Mean emergence time

Maize emergence was counted daily from three to 15 days after sowing at four randomly selected 3 m long row lengths. The values of mean emergence time (*MET*) was calculated following Celik and Alikat (2010) as in Equation (4),

(4) $$MET=\frac{\left(N_{1}T_{1}+N_{2}T_{2}+-----+N_{n}T_{n}\right)}{\left(N_{1}+N_{2}+----+N_{n}\right)}$$

where *N*_*1*_ … *n* is number of seedlings emerging since the time of previous count and *T*_*1*_ … *n* is the number of days after the sowing. The emergence rate index (*ERI*) and plant emergence degree (*PE*) were also following Equation (5),

(5) $$ERI=\frac{STE}{MET}$$

and Equation (6), respectively,

(6) $$PE=\frac{STE}{sn}\times 100$$

where *STE* is number of total emerged plants (seedlings) per meter, sn is number of seeds sown per meter.

#### 2.3.5 Seeding depth

At 30 days after sowing, maize seedlings were cut at the soil surface. The remaining portion of the stem left inside the soil (from soil surface to seed remnants on the root) was carefully removed and its length measured by caliper. Twenty randomly selected samples were taken from each plot.

### 2.4 Computations and data analysis

#### 2.4.1 Torque and power requirements for strip tillage

Power requirements for tillage varies by soil type, soil condition, operational speed, tillage depth, blade and furrow opener shape, and soil frictional characteristics (Kepner et al., 2005). Force exerted on blades during tillage was calculated using Equation (7),

(7) $$F=AxS_{p}$$

where *F* is draft force in N, *A* is soil area of cut by blades (cm^2^), and *S*_*p*_ is specific draft of the soil (N cm^−2^). Specific draft for clay loam soils was taken as 6 N cm^−2^ and 4 N cm^−2^ for loams following Kepner et al. (2005). Draft force is also inversely related to increasing soil moisture. In order to be conservative in our calculations, the maximum unit draft for clay soil was used to calculate force exerted on the blade.

The cross-sectional area cut by blades (*A*) was calculated using Equation (8),

(8) $$A=d\;x\;\omega \;x\;n_{bs}$$

where *d* is depth of cut (cm), *ω* is width of cut (cm), and *n*_*bs*_ is number of blade strikes at the same time. *d* was considered as 6 cm. When maize was strip-till seeded by PTOS, a maximum of one blades strike in the soil at a time. Width of cut varies depending on blade shape and was measured for each treatment. Torque (*T*, N-m) was determined for different blade shapes as in Equation (9),

(9) $$T=r_{b}\times F$$

where *r*_*b*_ is radius of blade tip rotation (m). Power (P) requirements (kW) for strip tillage was subsequently calculated using Equation (10),

(10) $$P=\frac{2\pi nT}{60\times 1000}$$

where *n* is blade speed (RPM) and *T* is torque.

#### 2.4.2 Statistical analyses

**Fig. 3:**
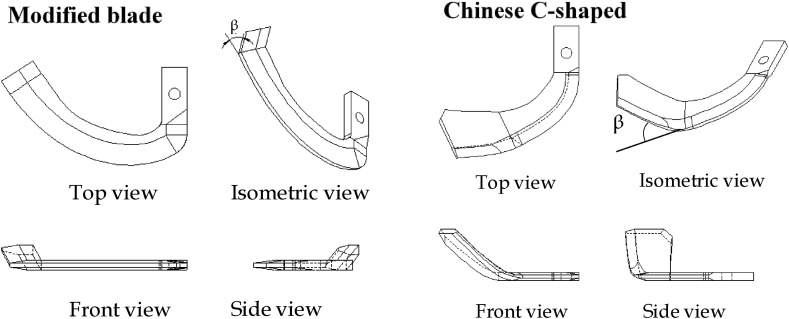
Orthographic projections of modified blades a with variable β tip rake angles (45°, 30°, 15° and 0°) ad Conventional C-shaped (47.67° angle) blades.

Conventional C-shaped blades were used as a control to contrast with the four modified blades which have a constant cutting edge curve angle. Angle tips (*β*) of the blades are shown in Fig. 3. Analysis of the variance (ANOVA) was performed considering season, RPM, blade design and repetition as fixed effects using JMP 8 software (SAS 9.4). Any non-significant interactions were removed in order up from three-to two-way interactions to improve power (Colegrave and Ruxton, 2017). Where significance was identified, means were separated using Tukey's Honestly Significant Difference *post-hoc* procedure.

**Fig. 4:**
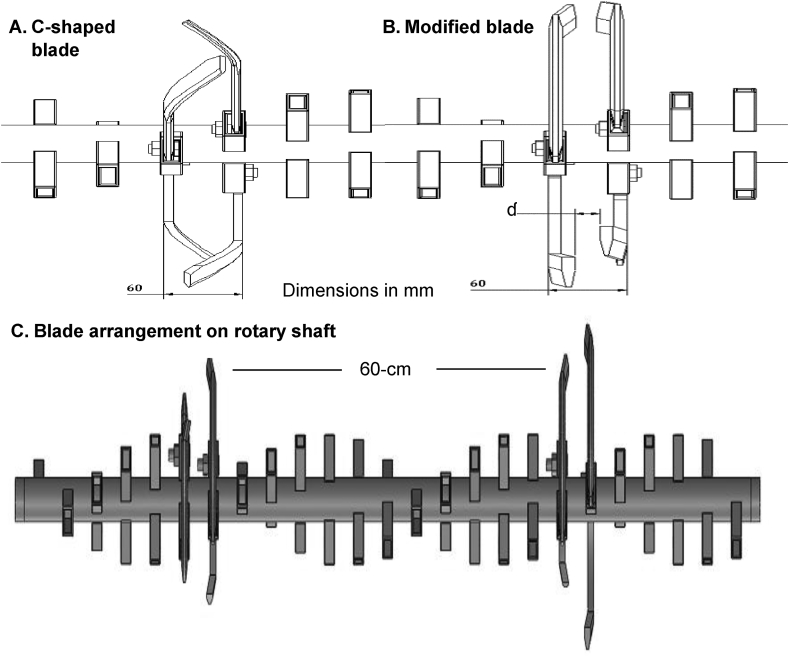
Different blade configurations showing arrangements on a rotary axle with detail of their and blade-to-blade interspace (ɗ). (A) C-shaped, (B) modified blade, and (C) blade arrangement on a two-wheel tractor-based power tiller operated converted seeder converted to strip tillage for maize by removing blades from their holders to facilitate a 60 cm row-to-row distance.

## 3 Results and discussion

### 3.1 Blade characteristics

Blade tip angle was the highest in the conventional blade and the lowest in modified blade with a 0° tip angle (Table 1). Length of the side section of the conventional blade was 65 mm, although it was 18 mm in all modified blades. The width of soil cutting ability was greater in the conventionally C-shape blade (46 mm) than modified blades with tip angles of 45°, 30°, 15° and 0° were 24.81, 20.47, 15.42 and 8.00 mm, respectively. The radius of all blades was 160 mm (Table 1).

### 3.2 Blade configuration and inter space

On a PTOS, shaft holders are fixed in a spiral configuration. During remounting of strip tillage blades, care was taken to arrange them face to face. Conventional C-shaped blades were spaced in such a way that width of cut of the right blade overlapped with the cut of the left blade (Fig. 4). The width of the modified blades was less than the C-shaped blades. Inter blade space therefore increased with decreasing tip angle. The inter blade spaces (*ɗ*) associated with 45°, 30°, 15° and 0° tip angles of the modified blades were 10.39, 19.06, 29.16, and 44 mm, respectively (Table 1). Strip tillage with a PTOS typically involves tilling soil and creating a narrow furrow <6 cm wide (Krupnik et al. 2013). Our design used an identical radius measurement, which rendered the blades compatible with the existing commercially available PTOS on which conventional C-shaped blades are typically used. The side-long length of modified blades was on average 72% less than the conventional C-shaped blade. The blade tip angle of modified blades (45°, 30°, 15° and 0°) reduced the blade width by 46, 56, 66 and 78%, respectively, compared to the C-shaped blade. Blade arrangement for strip tillage in the rotating shaft showed that the working width of the blades was constant (60 mm) for all blades. Considering the modified blades, the inter-blade free space increased as a result of the decrease in blades tip angles (Table 1).

### 3.3 Torque and power requirement for blade shapes and speeds

**Fig. 5:**
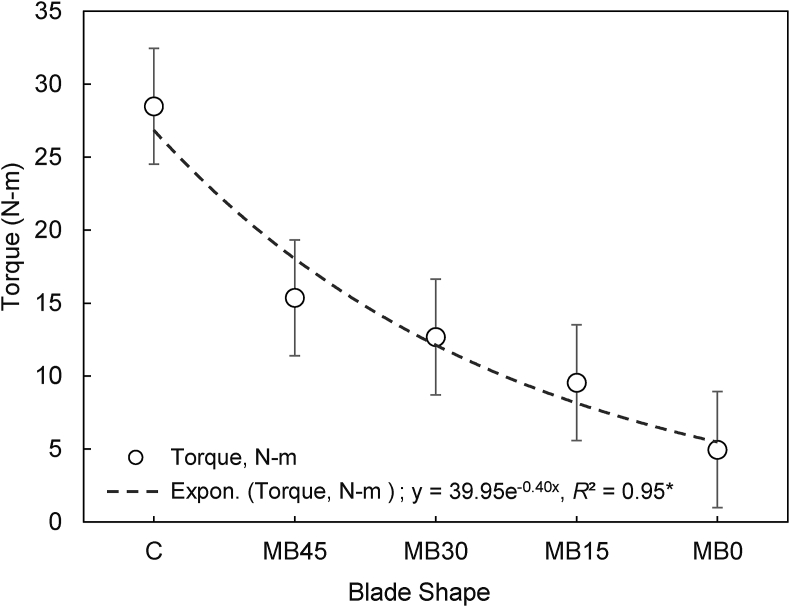
Estimated torque requirement for different blade shapes. C indicates the Chinese traditional blade, while MB indicates the modified blade with 45°, 30°, 15° and 0° tip rake angles. * indicates significance at *P* ≤ 0.05. Bars represent the standard error.

Strip tillage reduces the number of blades required in the rotating shaft of a PTOS. Two rows of maize seed can be planted with the common Chinese manufactured PTOS, using 2 × 4 = 8 blades for crops sown at 60 cm spacing. In our study, the blade holders on the rotary shaft were configured in such a way that a maximum of one blade struck the soil at the same time during strip till planting of maize. This reduced torque (Fig. 5). Torque requirement declined with decreasing blade tip angle. The highest torque was required for C-shaped blades (28.5 N-m). Within the modified blades, the lowest torque (4.96 N-m) was found with 0° angles and the highest (15.36 N-m) torque was associated with the 45° tip angle. Torque requirement was 63% higher for C-shaped blades than all modified blades, resulting in increased power requirements for furrow formation.

**Fig. 6:**
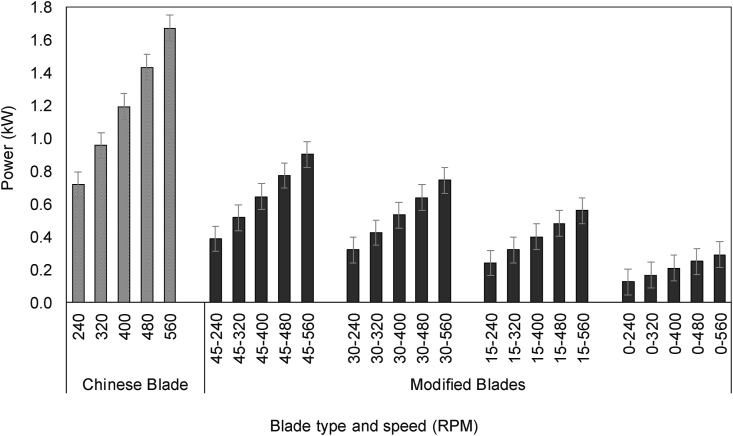
Estimated power requirements for different blade shapes and rotary speeds. C indicates the Chinese traditional blade, while MB indicates the modified blade with 45°, 30°, 15° and 0° tip rake angles. Numbers after dashes represent rotary speeds. Bars represent the standard error.

Power requirement was positively associated with increasing blade rotational speed blades, confirming observations made by Matin et al. (2015), Hassan (1980), and Niyamapa et al. (1994). More power was required for C-shaped than modified blades (Fig. 6). Within modified blades, 0° and 15° had similar power requirements at 320 RPM. Power requirements of modified blades with 15° and 0° tip angle for 320, 400 and 480 RPM rotational speed were also similar. The modified blade with a 15° tip angle for 320 RPM reduced the power requirement by 66% compared with the conventional C-shaped blades at 320 RPM (Matin et al., 2015). The torque and power requirement were directly influenced by soil type, blade shape, and rotational speeds of blade, the higher tip angle of the blade generated more torque and tillage resistance. These factors were also reflected in the observed higher power requirement (Kepner et al., 2005). Peak torque occurred at a higher blade speed (560 RPM) indicating transformation of the peak torque requirement from initial soil failure at a low speed, to final soil cutting and throwing at a high speed (Matin et al., 2015). The modified blade with minimum sidelong required less torque and power requirement. Similar results were also found for straight blade designs at 375–500 RPM by Matin et al. (2015). Increases in the blade tip length and angle to higher torque with the increasing rotary speed were also reported by Niyamapa and Chertkiattipol (2007).

### 3.4 Effect of blade shapes and speeds on furrow width

Furrow width was significantly higher in the first season when compared to the second season in both Jamalpur (Shown in Table 2) and Barishal (Table 3). In both locations, the widest strip furrow was found with 240 RPM and the narrowest with 560 RPM. In Jamalpur, similar strip furrow widths were found with 240, 320 and 400 RPM (61.88 mm). These were significantly (*P* = 0.01) wider than higher RPM of 480 and 560 (61.02 mm). In Barishal, furrow size followed the order of 240 RPM >320 RPM and 400 RPM >480 RPM and 560 RPM speed.

**Table 2:** Performance of different blade shapes and rotary speed for maize crop establishment in strip tillage at Jamalpur during 2013–15. C indicates the conventional C-Shaped blade, while MB indicates the modified blade with 45°, 30°, 15° and 0° tip rake angles.

Variables	Strip width (mm)	Cross sectional area (cm^2^)	Soil backfill (%)	Seed depth (cm)	Percent soil aggregates (1–8 mm)	Mean emergence time (days)	Emergence rate index	Plant emergence (%)
Maize growing season								
2013–14	62.40a	33.18a	60.75a	4.31	55.87 b	7.13	0.57a	81.10a
2014–14	60.67 b	31.36 b	54.56 b	4.35	59.76a	7.07	0.54 b	75.80 b

Speed of rotating blade (RPM)								
240	61.95a	31.30 b	55.48 b	4.35	57.07 b	7.16	0.55 ab	78.85 ab
320	61.75a	32.67 ab	61.09a	4.41	57.59 b	7.12	0.57a	81.60a
400	61.95a	31.61 ab	55.23 b	4.35	56.65 b	7.10	0.56 ab	80.02a
480	61.18 b	33.26a	59.11 ab	4.28	57.79 ab	7.08	0.55 ab	77.88 ab
560	60.85 b	32.53 ab	57.35 ab	4.27	59.96a	7.07	0.52 b	73.90 b

Blade shape (°angle)								
MB0	61.50	34.58a	63.84 ab	4.76 b	54.55 d	7.24a	0.58 b	83.25 b
MB15	61.58	34.57a	66.06a	4.93a	56.40cd	7.22a	0.62a	89.98a
MB30	61.50	33.55 ab	59.75 b	4.36c	57.64bc	7.13 ab	0.58 b	82.00 b
MB45	61.55	32.04 b	53.87c	3.93 d	58.98 b	7.01bc	0.50c	70.55c
C48	61.55	26.62c	44.73 d	3.67e	61.50a	6.92c	0.48c	66.47c

Analysis of variance								
Season (S)	<0.01*	<0.01*	<0.01*	0.23	<0.01*	0.12	<0.01*	<0.01*
RPM	<0.01*	0.02*	<0.01*	0.12	<0.01*	0.58	0.01*	<0.01*
Blade (B)	0.99	<0.01*	<0.01*	<0.01*	<0.01*	<0.01*	<0.01*	<0.01*
S × RPM	<0.01*	0.77	0.83	0.84	<0.01*	0.54	0.47	0.70
S × B	0.97	0.15	<0.01*	0.99	0.52	0.95	<0.01*	<.01*
RPM × B	0.48	0.99	0.99	<0.01*	0.99	0.99	0.46	0.42
S × RPM × B	0.97	0.99	0.84	<0.01*	0.99	0.99	0.44	0.39
Repetition (R)	0.20	0.54	0.10	<0.01*	0.93	0.34	0.46	0.21

Letters in columns not separated by a blank row followed by the same letter are not significantly different at alpha = 0.05 using Tukey's HST test. * indicates significance at 0.05. RPM = revolutions per minute.

**Table 3:** Performance of different blade shapes and rotary speed for maize crop establishment in strip tillage at Barisal during 2013–15. C indicates the conventional C-Shaped blade, while MB indicates the modified blade with 45°, 30°, 15° and 0° tip rake angles.

Variables	Strip width (mm)	Cross sectional area (cm^2^)	Soil backfill (%)	Seed depth (cm)	Percent soil aggregates (1–8 mm)	Mean emergence time (days)	Emergence rate index	Plant emergence (%)
Maize growing season								
2013–14	62.70a	32.17	56.33	4.22a	58.41a	7.10	0.54a	76.93a
2014–14	60.99 b	32.34	56.90	4.14 b	44.35 b	7.09	0.51 b	72.93 b

Speed of rotating blade (RPM)								
240	63.00a	31.43	55.72 b	4.16 ab	48.59 b	7.11	0.52	73.58
320	62.35 b	32.83	59.21a	4.25a	50.81 ab	7.14	0.54	77.58
400	62.03 b	32.07	56.57 ab	4.22 ab	51.93a	7.12	0.54	76.58
480	61.10c	32.67	56.32 ab	4.13 ab	52.21a	7.04	0.52	73.67
560	60.75c	32.27	55.26 b	4.12 b	53.36a	7.06	0.52	73.25

Blade shape (°angle)								
MB0	61.70	33.52a	62.68a	4.53 b	48.48c	7.21a	0.56 b	80.75 b
MB15	61.85	34.05a	65.07a	4.76a	49.98c	7.20a	0.60a	86.08a
MB30	61.85	33.15 ab	59.00 b	4.20c	51.02bc	7.14 ab	0.53bc	75.67c
MB45	61.93	31.80 b	52.06c	3.83 d	53.14 ab	6.98bc	0.50c	69.08 d
C48	61.90	28.75c	44.27 d	3.58e	54.27a	6.94c	0.46 d	63.08e

Analysis of variance								
Season (S)	<0.01*	0.61	0.45	<0.01*	<0.01*	0.81	<0.01*	<0.01*
RPM	<0.01*	0.05	0.01*	0.016*	<0.01*	0.67	0.15	0.03*
Blade (B)	0.82	<0.01*	<0.01**	<0.01*	<0.01*	<0.01*	<0.01*	<0.01*
S × RPM	0.03*	0.33	0.51	1.00	0.69	0.98	0.90	0.89
S × B	0.99	0.01*	<0.01*	0.09	0.64	0.93	0.02*	0.03*
RPM × B	0.06	0.78	<0.01*	<0.01*	0.88	0.98	0.97	0.89
S × RPM × B	0.99	0.15	0.02*	0.08	0.85	0.99	0.97	0.87
Repetition (R)	0.50	0.82	0.49	0.16	0.24	0.59	0.30	0.19

Letters in columns not separated by a blank row followed by the same letter are not significantly different at alpha = 0.05 using Tukey's HST test. * indicates significance at 0.05. RPM = revolutions per minute.

A significant interaction (*P* = 0.03) between season and rotary speed was observed on strip furrow width at Jamalpur and Barishal (Table 4). In both locations, the widest strip was found with 240 and 320 RPM in the first season and the narrowest with 560 RPM in the second season. In both seasons, the widest (240 RPM) and narrowest (560 RPM) strip widths were significantly different (P = 0.01). Similar results were also reported by Celik and Altikat (2008). Interestingly, no differences in strip width at 560 RPM were found in the first season when compared with 240 RPM in the second season. Strip width depended on the soil moisture content. Similar findings also were observed elsewhere, i.e., low soil moisture and high rotational speed increases furrow width (Lee et al., 2003). In the first season, wider strip width was associated with lower soil moisture content (25.31%) than in the second season (27.01%). This may be because of higher crop residue was retained in field in the first season (2.5 t ha^−1^) than the second season (2.0 t ha^−1^). Other studies have also indicated that soil moisture content influences the cutting width of blades by creating more resistance and ultimately soil displacement (Lee et al., 2003).

**Table 4:** Interactive effect of season and rotary speed of blades (240, 320, 400, 480, 560 RPM) on **Furrow Width** of maize drilled by strip tillage in Jamalpur and Barisal in Bangladesh during 2013–14 and 2014–15.

Year	Rotary speed (RPM)	Jamalpur	Barishal
2013	240	63.25a	64.25a
	320	62.75a	63.00 b
	400	62.65 ab	62.90 b
	480	61.85bc	61.85c
	560	61.50cd	61.50c
2014	240	60.65 def	61.75c
	320	60.75 def	61.70c
	400	61.25cde	61.15cd
	480	60.50ef	60.35de
	560	60.20f	60.00e

Letters in columns not separated by a blank row followed by the same letter are not significantly different at alpha = 0.05 using Tukey's HST test. * indicates significance at 0.05. RPM = revolutions per minute.

### 3.5 Effect of blade shapes and speeds on furrow cross-sectional area

Measured cross-sectional areas of the furrows were significantly higher in the first season when compared to the second season at Jamalpur (Table 2), but not in Barishal (Table 3). In the former location, the narrowest cross-sectional area was recorded with 240 RPM and highest was with 480 RPM at Jamalpur. The narrowest cross-sectional area was found with C-shaped blade than all modified blades, with conventional blades resulting in 26.5% and 15.2% narrower cross-sections in Jamalpur and Barishal, respectively. We also observed that the 45° tip angle blade produced lower cross-sectional area the 0° tip angle in both locations. Greater cross-sectional area was found with 0° and 15° modified blades in both locations, while 30° had intermediate effects within modified blades.

The cross-sectional area of the furrow depends on blade geometry and rotating speed. Cross-sectional area varied as a result of the different shapes of blades tested. Though rotating blade diameter was similar among the modified blades, the effective cutting area was different depending on the geometry of the blade. Cutting area varied mainly as a result of the length of the sidelong section and the tip angle. Cutting width of the C-shaped blade was greater than the modified blades, but overall cutting area was smaller. The cutting width of the modified blades was inversely related to the blade tip angle, an observation that affected the cross-sectional area of the strip. Cross-sectional areas in the first season were found higher than that of the second season and this was likely to have been caused by higher crop residue cover as a mulch in the first season. In Jamalpur, a smaller cross-sectional area was found with lower blade rotary speeds similar to normal power tiller speed (240 RPM). Higher speeds however resulted greater cross-sectional areas. Such increase can be attributed to variation of the furrow bottom (Celik and Altikat, 2008), as well through an overall improvement in uniformity of the furrow wall shape. Matin et al. (2014) also suggested that the volume of furrow in strip tillage can be significantly affected by both blade geometry and rotary speed, including conventional C-shape and modified blades.

### 3.6 Effect of blade shapes and speeds on soil backfill

No differences in soil backfill were found in Barishal. Considering Jamalpur, however, backfill was significantly (*P* < 0.001) greater in the first season than the second season. Backfill tended to be greatest with a blade speed of 320 RPM (61.1% and 59.2% at Jamalpur and Barishal, respectively). Lower backfill was observed with 560 RPM in both Barishal and Jamalpur (Table 2, Table 3). The lowest soil backfill was measured with C-shaped blades, whereas greater backfill was observed with the modified 15° and 0° tip angle in both locations. The 45° tip angle provided better soil backfill when compared to the C-shape blade, though it was inferior other modified blade shapes in both locations.

A significant interaction (P = 0.01) of blade shapes and rotary speed of blades on soil backfill of furrow was observed in Barishal (Table 5). Soil backfill for the interactive effect of modified blades with 0° and 15° tip angles across all RPMs only showed difference in between 240 RPM at 0° and 320 RPM at 15°. Similarly, the 30° and 45° tip angle blades showed no differences except for 240 RPM with the 45° tip. The effect of the conventional C-shaped blade across rotational speed was the lowest than modified blades except 240 RPM at 45° and C blade.

**Table 5:** Interactive effect of blade shapes and rotary speed of blades (240, 320, 400, 480, 560 RPM) on percentage **Soil Backfill** and **Seed Depth** of maize drilled by strip tillage in Barisal and Jamalpur in Bangladesh during 2013–14 and 2014–15.

Blade shape	Rotary speed (RPM)	Barishal		Jamalpur
		Percentage soil backfill	Seed depth (cm)	Seed depth (cm)
MB0	240	57.18bcd	3.94efg	4.61 abcd
	320	66.86 ab	4.72 ab	4.90 ab
	400	63.99 abc	4.61 ab	4.59abcde
	480	63.37 abc	4.71 ab	4.88 ab
	560	62.02 abcd	4.67 ab	4.84 abc
MB15	240	63.58 abc	4.57 abc	4.86 abc
	320	69.20a	4.83a	5.01a
	400	64.84 ab	4.82a	4.87 abc
	480	64.08 abc	4.79 ab	4.98a
	560	63.66 abc	4.77 ab	4.94 ab
MB30	240	58.77bcd	4.45bcd	4.49bcde
	320	60.57 abcd	4.24cde	4.40cdef
	400	59.37bcd	4.19de	4.49bcde
	480	58.69bcd	4.00efg	4.15defgh
	560	57.61bcd	4.13de	4.28defg
MB45	240	46.39efg	3.76 fgh	3.96 fghi
	320	54.66cde	3.98efg	4.13efgh
	400	52.90 def	4.00efg	3.94 fghi
	480	53.57 def	3.75 fgh	3.86ghi
	560	52.76 def	3.65gh	3.78hi
C48	240	52.68 def	4.09ef	3.84 ghi
	320	44.74 fg	3.46 h	3.58i
	400	41.76 g	3.51 h	3.86ghi
	480	41.90 g	3.42 h	3.55i
	560	40.26 g	3.41 h	3.53i

Letters in columns not separated by a blank row followed by the same letter are not significantly different at alpha = 0.05 using Tukey's HST test. * indicates significance at 0.05. RPM = revolutions per minute; C indicates the Chinese traditional blade, while MB indicates the modified blade with 45°, 30°, 15° and 0° tip rake angles.

**Fig. 7:**
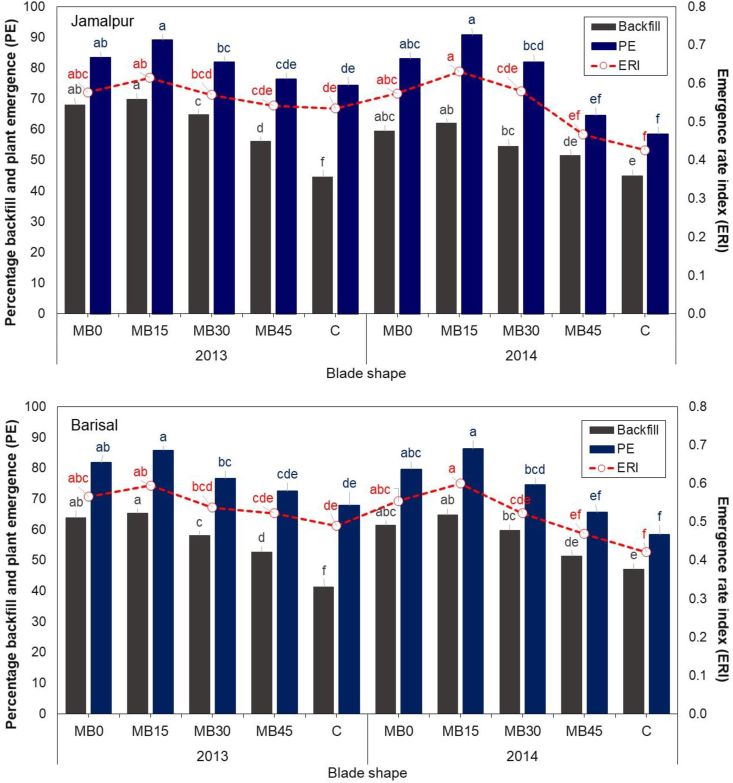
Interactive effect of season and blade shapes on the percentage of soil backfill, plant emergence (PE) and emergence rate index (ERI) at Jamalpur and Barisal in Bangladesh during 2013–14 and 2014–15. C= Chinese traditional blade. C indicates the Chinese traditional blade, while MB indicates the modified blade with 45°, 30°, 15° and 0° tip rake angles. Different letters for matching columns or dotted lines within same color represent to significance at *P* ≤ 0.05.

A significant interaction (*P* = 0.043) was found between season and blade shape on the percentage of soil backfill at Jamalpur and Barishal (Fig. 7). The same trend was followed for interaction effects in both the locations. The lowest backfill in the first season and also second season was observed with the C-shaped blade but former season was inferior than later. The greater soil backfill of furrow was observed in first season at 15° which was significant to two other modified blade at 30° and 45° in both seasons.

Soil backfill appears to have been influenced by blade geometry and the operational speed of the rotating blades, in addition to soil type. The C-shaped blade produced the least soil backfill in the furrows in both locations. As with other parameters, soil backfill modified blades varied with blade tip angle, with the greater angle producing poorer backfill and hence seed coverage in furrows in both locations. Straight blades and the modified blade with a 15° tip angle produced similar volumes of soil backfill, although straight blades sometimes failed to pulverize the soil when moisture was high, particularly in the heavy clay soil found in Barishal. Soils in Barishal also had higher clay content than Jamalpur. Under these conditions, the modified straight blade was unable form strip furrows appropriately. Matin et al. (2014) also found the greatest volume of soil backfill when straight blades were used for strip tillage, but on a coarser textured soil. Beeny and Khoo (1970) suggested the degree of a blade's bend width can influence soil carrying, throwing, and pulverization. They also found that higher and lower speeds both resulted in poor soil backfill when compared to recommended rotational speed. These parameters affect the amount of soil remaining in furrows under strip tillage, and are therefore important parameters in seedbed formation and germination potential. In our study, the greatest soil backfill was found with 320 RPM when an inverted-T furrow opener was used. This observation backs Matin et al. (2014), who reported no significant decrease in backfill with increasing speed above 250 to 500 RPM for straight blades, and 375 to 500 RPM for conventional and half width blades.

### 3.7 Seeding depth of maize

Seed depth of maize at Jamalpur was similar in both seasons (Table 2). At Barishal, however, depth was significantly (*P* = 0.01) greater higher in the first season compared to the second (Table 3). Maize seeding did not vary as a function of RPM at Jamalpur, differences in depth were found in Barishal. The deeper maize seeding depth was associated with 320 RPM than 560 RPM. Maize seeding was also influenced by blade shapes at both locations. The deepest seeding was found with the modified blade 15° tip angle followed by 0°, 30° and 45° tip angles. The shallowest seeding was associated with C-shaped blades in both locations.

A significant (*P* = 0.01) interaction between blade shapes and rotary speeds were found to affect maize seeding depth in Jamalpur and Barishal (Table 5). In both locations, the combination of 15° tip angle at 320 RPM gave significant deeper maize seed depth than 30° and 40° tip angles at all rotations. The shallowest seeding depths were found with C-shaped blade at all rotational speeds than modified blades except 45° tip angle where few seeding depth variations were also noticed in 45° tip angle with C-shaped blades with different rotary speeds at both locations.

Seeding depth was greater when furrows were formed using modified blades with reduced tip angles. This effect was likely caused by greater soil backfill than achieved with the conventional blade. With similar clay loam soils in Turkey, Ozmerzi et al., 2002 examined the effect of sowing maize at different depths and suggested 60 mm as an ideal sowing depth in terms of vertical seed distribution uniformity. Our results appear to support this finding for modified blades with 15° tip angle. Insufficient seed soil coverage and soil-to-seed contact results in poor germination, though overly deep seeding can also delay emergence (Loeppky et al., 1989). Consistent sowing depth is an important parameter to achieve uniform crop emergence (Choudhary et al., 1985; Stockton et al., 1996), and Desbiolles (2004) reported that the sowing depth is dependent on both the physical placement of seeds in the furrow and the amount of soil cover subsequently added. Our findings therefore provide support for these observations, but also advance knowledge with respect to seeding depth and furrow in-fill under strip tilled maize in rice-maize cropping systems of the tropics.

### 3.8 Effect of shapes and speeds of the blade on soil aggregates

Patterns of soil aggregation were significantly (*P* = 0.01) different between seasons in both locations. At Jamalpur, the percent of desirable soil aggregates that were between 1 and 8 mm in diameter was greater in the first season when compared to the second season. In Barishal, the opposite pattern was observed. Previous research has found that increasing rotational speed of tillage blades can decrease range of desirable soil aggregate sizes (Khan et al., 2015; Hashemi et al., 2012). At Barishal, the lowest percentage of desirable soil aggregates were observed with 240 RPM, above this, aggregates were greater than 8 mm, with the exception of 320 RPM.

The greatest percentage of soil aggregates within the desirable 1–8 mm range (cf. Celik and Alikat, 2010) was found with C-shaped blades in both locations, though similar amounts were also found with the 45° tip angle modified blade at Barishal. Lower percentages of desirable soil aggregate sizes were found with modified 0° and 15° tip angle blades at Jamalpur, and with the modified 0°, 15° and 30° tip angle at Barishal. Modified blades with 30° and 45° tip angles tended to produce similar amounts of aggregates in the 1–8 mm range.

The literature indicates that soil aggregate size ranging from 1 to 8 mm diameter tends to be ideal for seedbed formation (Celik, 1998). Differences in the desired soil aggregate range were found depending on the season of experimentation, and can be lined to soil moisture variability, in addition to blade geometry and speed (Naderloo et al., 2009; Olatunji, 2007). Crucially, although we found the greatest percentage of optimally sized soil aggregates with the C-shaped blade, recovered soil aggregates were located on the soil surface after having been thrown from the strip furrow, ultimately resulting in a poor seedbed. The modified blades for strip tillage with 0° and 15° tip angles produced similar quantities of soil aggregates of the desired diameter range. More than 50% of displaced soil aggregates were found in the desired size range of 1–8 mm with these blades. Yet among these blades, the 15° tip angle blade ensured the greatest backfill.

### 3.9 Mean emergence time

There was no difference in mean emergence time (MET) of maize between seasons regardless of the speed of rotating blades in both locations. MET however varied significantly as a function blade shape. The greatest mean emergence time was found with modified blades with 0° and 15° tip angles in both locations. The conventional C-shaped and 45° angled modified tip blades were similar, while the 30° tip angle modified blade resulted in intermediate MET. Mean emergence time was also longer for the modified blades in comparison to the conventional C-shaped blade as a control. This resulted from the greater seeding depth observed with the modified blades and greater backfill, supporting research by Loeppky et al. (1989) and Alikat (2013). In addition to sowing depth, MET can also vary with seed size. Soil temperature, topsoil crusting, and soil to seed contact, in addition to factors such as seed movement from attempted predation (Morrison and Gerik, 1985; El-Abady, 2015).

### 3.10 Emergence rate index

Emergence rate index (ERI) was greater in the first season compared to the second season in both Jamalpur and Barishal. ERI varied as a result of rotating blade speed at Jamalpur, though no differences were found in Barishal. Maximum ERI was found with 320 RPM and 560 RPM but other speeds resulted in no differences.

Blades shape also affected ERI in both experimental locations. The greatest ERI was found when sowing was completed using the modified 15° tip angle blade – an observation that held constant in both Jamalpur and Barishal. In the former location, a lower ERI was however also found when using the C-shaped blade and modified 45° tip angle blade. In Barishal, with modified 45° and 30° tip angle blades also resulted in lower ERIs.

Significant differences and an interactive effect of season and blade shapes was found to affect the ERI in both Jamalpur and Barishal (Fig. 7). A combination of modified blade 15° tip angle and season two gave significant greater ERI at both locations except 0° and 15° tip angle in season one and 0° tip angle in season two. On the other hand, the C-shaped blade in both seasons gave the lowest ERI compared to all modified blades except 45° tip angle in both seasons. Application of 240–480 RPM had no effect on ERI, but 560 RPM resulted in poorer soil backfill and shallower seeding depth, negatively affecting ERI. The modified 15° tip angle blade conversely resulted in deeper seed drilling and improved soil back fill. Though the seeding depth was shallower with the C-shaped blade, emergence uniformity and hence the crop stand was poor.

### 3.11 Maize emergence

Germination percentage and emergence rate are typically positively associated (Alizadeh et al., 2013). The greatest percent of emerged maize plants was found in the first season (77–81%) in comparison to the second (73–76%) (Table 2, Table 3). Emergence also appears to have been influenced by blade RMP in Jamalpur, not in Barishal, indicating the inconsistent influence of blade rotational speed on plant emergence. The greater and smaller plant emergence was associated with 320 RPM (81.60%) and 560 RPM (73.90%). The greatest (86–90%) and lowest (63–77%) maize emergence was found with the modified 15° tip angle and C-shaped blade at both locations. Sowing depth < 4 cm can result in a number of negative effects on overall crop stand. For example, shallower than ideal nodal root development can cause maize to be prone to early-season root lodging. Seedlings can also be more prone to injury where farmers apply pre-emergent herbicides (Luce, 2016).

Interaction of season by blade shapes had significant influence on plant emergence at both Jamalpur and Barishal (Fig. 7). In both seasons, the plant emergence was significantly larger with 15° tip angle compared to the rest of the blade shapes except 0° tip angle. On the other hand, the lowest plant emergence was associated with the interactive effect of second season and C-shaped blade but this was similar to the second season at modified blade 45° tip angle.

Maize emergence responded variably to blade rotational speed and design. The 320 RPM case resulted in the greatest soil backfill and seeding depth, as well as greatest crop emergence. The modified 15° tip angle blade also resulted in higher soil backfill and seed depth, which in turn also appears to have affected emergence. Uneven soil moisture in the seedbed is a primary cause of uneven emergence. An optimum seeding depth of 5–6 cm was recommended by Hussen et al. (2013) and Aikinss et al. (2006). In the current study, sowing depth >8 cm was associated with poor emergence. This is potentially because the apical shoot may encounter difficulty in pushing through the soil when seeds are sown at depth, thereby negatively affecting crop stands (Hussen et al., 2013). Greater soil backfill in strip tillage can assist in improving emergence, and the 15° tip angle blade and 320 RPM speed appear to have been influential in germination in both experimental locations. These results however should be treated with caution as farmers in different environments and with different top soil moisture content will need to adjust seeding depth to correspond with the appropriate level of moisture to encourage germination.

## 4 Conclusions

We examined the implications of modified strip tillage blade designs on furrow architecture, seed bed characteristics, and maize crop establishment over two cropping seasons at two locations with differing soil types in Bangladesh. Use of modified blades resulted in improved seedbeds with a higher volume of soil backfill than the widely available conventional C-shaped blades that come with Chinese manufactured commercially available power tiller operated seeders. Torque and power requirements were also associated with increased in blade tip angles, although all modified blades had lower power requirements than the C-shaped blade. Conventional C-shaped blades produced the largest volume of desirably sized soil aggregates, although these were thrown outside the furrow onto the soil surface. Conversely, the modified 30°, 15° and 0° tip angle blades produced similar volumes of soil aggregates in the desired diameter range. Our analysis indicates that condition of 320 RPM with the modified 15° tip angle blade are likely to result in optimal furrow cross-sectional area, producing the greatest soil backfill and uniform seed depth with better soil-to-seed contact to achieve optimum crop establishment. This configuration also tended to result in improved soil backfill with minimum soil throwing and optimum soil aggregate size, resulted in the greatest seed depth, minimum MET, higher ERI and higher plant emergence.

In Bangladesh, both smallholder farmers and mechanized service providers understand the benefits of CA-based strip tillage and mechanized seeding, in particular for the cultivation of dry season winter crops following wet season ‘*aman*’ rice. However, farmers and service providers regularly face challenges with existing mechanical seeders in terms of poor and uneven crop establishment. With current machinery configurations, mechanized seeding results in poor seeding depth, insufficient coverage of the seed in the furrow after planting, drying of seeds after planting leading to poor seed germination, and seed predation by birds of poorly covered seeds. A mechanized seeder with an improved blade design used at an appropriate speed will be immediately attractive to service providers and farmers, and increase the demand for locally manufactured two-wheel tractor attachments such as seeders or strip tillage planters with modified blades. This has the potential to increase the scale of adoption of CA-based mechanized seeding and to sustainably improve both crop production and service provision. This configuration thus appears to be a robust alternative to conventional blades that can be used to improve strip tillage crop performance in Bangladesh and other locations with similar rice-based rotations and soil textures.

## Declaration of competing interest

The authors declare that they have no known competing financial interests or personal relationships that could have appeared to influence the work reported in this paper.
